# Influence of multi-dimensional environmental knowledge on residents' waste sorting intention: Moderating effect of environmental concern

**DOI:** 10.3389/fpsyg.2022.957683

**Published:** 2022-11-29

**Authors:** Zhihua He, Yong Liu, Xiaochun Liu, Feng Wang, Huijia Zhu

**Affiliations:** College of Management Science, Chengdu University of Technology, Chengdu, Sichuan, China

**Keywords:** waste sorting intention, system knowledge, action-related knowledge, effectiveness knowledge, environmental concern

## Abstract

With the rapid increase in household waste, environmental degradation becomes more serious. It is imperative to promote waste sorting in China. This study proposes an extended KAB model to explore the impact mechanism of different dimensions of subjective environmental knowledge on urban residents' waste sorting intention. The study also explores the moderating role of environmental concern in the relationship between three types of subjective environmental knowledge and attitude toward waste sorting. Based on 308 valid questionnaires, through structural equation model, multiple regression analysis, and simple slope test, we found that system knowledge, action-related knowledge, and effectiveness knowledge all have positive impacts on residents' attitudes toward waste sorting, and effectiveness knowledge has the most significant impact. Meanwhile, environmental concern positively moderates the relationship between system knowledge, effectiveness knowledge, and attitude toward waste sorting. This study makes an important theoretical contribution to enrich the existing literature on residents' waste sorting behavior and provides theoretical insights for governmental waste sorting policy formulation at the practical level.

## Introduction

With the rapid development of the global economy and urbanization, municipal solid waste has increased at a high rate of speed around the world. It is estimated that global waste production will increase from 2 billion to 3.5 billion tons in the next 30 years (Lin et al., 2017; Wang et al., 2019). A large amount of waste has caused severe environmental pollution and threatened the sustainable development of society and human health (Wang S. et al., 2020). For example, waste disposal leads to the occupation of large land resources and pollution of groundwater, soil, and air (Li et al., 2016; Wang et al., 2019). As a country with a large population, China also has the heaviest waste burden in the world. Accompanying rapid population growth, urbanization, and industrialization, China's total waste production has increased significantly, and China has become the world's leading waste producer (Tong et al., 2020). In 2014, China's total household waste had risen by 178.6 million tons, with an annual growth of about 1.6% between 2005 and 2014 (National Bureau of Statistics of China, 2018). In addition, 85% of China's waste is disposed of in landfills (Huang and Yang, 2013). Landfills without proper classification will cause soil and groundwater pollution (Zhang et al., 2019). Obviously, the accumulation of urban waste brings great challenges to urban environmental management. However, compared with landfill and incineration disposal in China, the classification rate of domestic waste is relatively low (Zhang et al., 2016; Wang et al., 2020). Among these phenomena, the source sorting of waste is not only the premise but also the key. Waste sorting engagement is personal and related to environmental attitudes, including a willingness to sort, ecological concerns, a perceived moral obligation, and a sustainability attitude (Liu et al., 2019; Zhang et al., 2019; Shan et al., 2020). Meanwhile, Chen and Gao (2020) highlighted that waste management's success depends on residents' engagement in classification activities. Public participation in waste sorting is considered an important part of the waste management chain (Tong et al., 2020; Hu et al., 2021). How to improve the residents' participation rate is an urgent problem to be solved (Meng et al., 2019). China's waste sorting remains low, making it imperative to research and provide scientific policy recommendations to promote waste sorting (Hongping et al., 2020). Therefore, it is of great theoretical and practical significance to explore in depth the main factors influencing residents' domestic waste disposal behavior and then to develop targeted urban waste management policies to increase the participation rate of residents in sorting and recycling (Meng, 2019). In the early stages, most studies on the factors influencing household waste sorting behavior focused on external conditions such as policy measures and convenience (Wertz, 1976; Jenkins and Jarvinen, 1995; Fullerton and Kinnaman, 1996; Linderhof et al., 2001; Domina and Koch, 2002; Dijkgraaf and Gradus, 2004; Callan and Thomas, 2006; Iyer and Kashyap, 2010; Bernstad, 2014; Xu et al., 2017). Some researchers also believe that household waste sorting behavior is the consequence of residents' active selection, and the key point is the actor itself. Sequentially, they begin to focus on the internal characteristics of individuals (attitude, responsibility, perceived behavior control, etc.). In fact, the public's understanding of waste sorting and recycling is the first step toward their independent waste separation and rational use of resources (Almasi et al., 2019). Thus, environmental knowledge is considered to be a crucial predictor of household waste sorting behavior. The theory of reasoned action (TRA) has been widely used in behavioral research due to its great applicability to environmental psychology (de Leeuw et al., 2015; Echegaray and Hansstein, 2017; Oztekin et al., 2017; Cudjoe et al., 2020; Han and Cudjoe, 2020). The TRA has been used as the basic framework in most of the studies related to environmental knowledge (Ajzen and Fishbein, 1980), where the attitude and behavior variables in the TRA are taken out separately (Polonsky et al., 2012; Babaei et al., 2015; Taufique et al., 2016; Baser et al., 2017; Almasi et al., 2019). As a result, a new model that only includes knowledge, attitude, and behavior (KAB) is gradually formed (Polonsky et al., 2012; Babaei et al., 2015; Taufique et al., 2016; Baser et al., 2017; Almasi et al., 2019). Baser et al. (2017) studied the relationship between food safety knowledge, food safety attitude, and food safety behavior. Practices are the community's actions affected by knowledge and attitude (Kofoworola, 2007; Knickmeyer, 2020). Citizens of developing countries are mostly not involved in waste management decision making, which in turn reduces their concern, attitude, and practice toward waste recycling and management programs (Essuman, 2017). Countries such as Iran have not gained significant achievements in waste sorting, not only because of the financial and technical limitations but also because of the lack of adequate public awareness and attitude in this context (Zand et al., 2020). Therefore, in this study, the KAB model is used as the fundamental theoretical framework for exploring the impact of environmental knowledge on domestic waste sorting behavior. Besides, environmental knowledge is mostly studied as a single dimension in this model. However, environmental knowledge is considered to be a complex and multi-dimensional factor rather than a single and coherent one (Kaiser and Fuhrer, 2003). Therefore, a more comprehensive classification of environmental knowledge in the KAB model is necessary.

Many scholars have divided environmental knowledge into subjective and objective knowledge (Ellen, 1994; Carmi et al., 2015; Onel and Mukherjee, 2016; Casaló et al., 2019). In the literature about subjective and objective knowledge, a lot of research has confirmed that the former has a greater impact on personal environmental behavior (Ellen, 1994; Kaiser and Fuhrer, 2003; Aertsens et al., 2011; Casaló et al., 2019). Although subjective knowledge and objective knowledge are two dimensions of environmental knowledge, they are divided according to the environmental actors themselves. This classification method also fails to comprehensively analyze the relative influences of multiple knowledge of environmental protection behaviors, and that leads to an inadequate understanding of how various types of knowledge affect environmental protection behaviors (Frick et al., 2004). Therefore, in the context of waste sorting, it is still necessary to study the influence of different forms of subjective knowledge on residents' waste sorting behaviors.

In addition, Tadesse (2009) pointed out that environmental concern is closely related to environmental knowledge, and their relationship needs to be further verified. It has been verified that a certain connection exists between environmental concerns and environmental knowledge. Das and Ramalingam (2019) studied how environmental concern plays a moderating role in the influence of people's perceived environmental knowledge on price equity. According to Das and Ramalingam (2019), it can be speculated that a moderating relationship may also exist between environmental concern and subjective environmental knowledge. Besides, in the studies of Braun and Dierkes (2017) and Casaló et al. (2019), there was only a weak correlation between environmental knowledge and attitude. From this, it can be further speculated whether environmental concern can enhance the relationship between them. Therefore, it is necessary to incorporate environmental concerns into the KAB model and further verify its regulatory role.

Therefore, in order to fill these gaps, this study proposes an extended KAB model. It includes the antecedent factors of attitude, namely, three types of subjective environmental knowledge. Besides, adding environmental concern will moderate the impact of antecedent factors on attitude, so as to further study domestic waste sorting behavior.

This paper enriches the existing literature on the study of domestic waste sorting behavior. First, it provides a new research model for waste sorting. Second, it confirms the different degrees of facilitation of three different dimensions of subjective environmental knowledge, which contribute to waste sorting intentions through attitudes toward waste sorting. Third, the findings of this paper also provide a theoretical reference for the government to formulate relevant policies on waste sorting.

The next arrangement of this paper is as follows: First, the theoretical background of residents' waste sorting behavior is introduced; second, the research model and hypotheses of this paper are presented; third, the research methods and results are described; and finally, the theoretical and managerial significance of this article and the future research directions in this field are discussed.

## Literature review and hypotheses

### Waste sorting

From now on, most scholars have studied the affecting factors of household waste sorting behavior from two perspectives. One is the internal factor (Xu et al., 2017), which means that the environmental choice behavior is the “active choice” of the actor and the inherent characteristics of the behavior subject. The other is the external factor (De Feo and De Gisi, 2010; Xu et al., 2017), which refers to the external environmental factors that promote actors to classify waste, including classification services and facilities, social norms, and promotion measures. Most of the current research has focused on external factors. For instance, scholars such as Linderhof et al. (2001), Dijkgraaf and Gradus (2004), and Callan and Thomas (2006) extended Wertz's (1976) research to reveal the direct and pronounced effects of incentives and penalties on waste sorting behavior. Iyer and Kashyap (2010) further demonstrated the timeliness of the incentive policy and believed that the policy effect would disappear with the end of the policy. Xu et al. (2017) further divided incentives into market and government incentives, which proved that government incentives, government promoters, and market promoters had significant positive effects on residents' recycling behavior. In addition to the study of incentive measures, the convenience of waste sorting is also the focus of scholars. For example, Domina and Koch (2002) pointed out that convenience is a significant trigger event for textile recycling behavior. Bernstad (2014) also stressed the importance of the necessary infrastructure for accessible and convenient domestic waste classification.

Although these studies provide useful insights for understanding waste sorting behavior, it is more important to know the significance of the psychological factors affecting residents' waste sorting activities (Babaei et al., 2015). Some scholars believe that knowledge is an important psychological factor affecting residents' waste sorting behavior. For instance, Bortoleto et al. (2012) argued that personal knowledge was regarded as an important and influential factor in practice, which greatly promoted the implementation of recycling programs and ensured their success. Choudri et al. (2016) also argued that understanding public awareness and perspectives on environmental issues in specific areas is an important part of promoting public pro-environmental behavior. This means that knowledge is a major and fundamental element of the study on household waste sorting behavior. Among the existing studies in which knowledge is the main factor, most of them take knowledge, attitude, and behavior (KAB) as the research model (Polonsky et al., 2012; Babaei et al., 2015; Taufique et al., 2016; Baser et al., 2017; Almasi et al., 2019; Khan et al., 2021). In the literature mainly based on research knowledge, the model has better interpretation and prediction abilities. Although this model adequately reveals how individual environmental knowledge affects environmental behavior, it also has some shortcomings, such as the wide coverage of environmental knowledge. Therefore, a new model is needed to further explore the residents' waste sorting behavior.

### Modified knowledge, attitude, and behavior model

Fishbein and Ajzen established the theory of reasoned action (TRA) to forecast human actions under full-purpose regulation (Untaru et al., 2016). In this theory, a person's willingness to participate in a specific action is obvious from their intention, which is the direct premise of the action (Cudjoe et al., 2022). Researchers have widely used the TRA as a means to grasp the relationship between knowledge level and actual behavior (Polonsky et al., 2012; Paco and Lavrador, 2017; Zheng et al., 2021). In terms of waste management, some studies have applied the TRA to explore waste sorting intentions (Cudjoe et al., 2020) and waste separation (Oztekin et al., 2017). With the in-depth study of knowledge, we constructed an extended TRA model, the knowledge, attitude, and behavior (KAB) model (Polonsky et al., 2012; Babaei et al., 2015; Taufique et al., 2016; Baser et al., 2017; Almasi et al., 2019). This model explains how knowledge affects attitudes, which in turn affects behavior indirectly (Polonsky et al., 2012). Arcury (1990) first concluded in his research that the environmental attitudes and intentions of North American consumers can be positively influenced by their environmental knowledge. Bang et al. (2000) argued that environmental knowledge positively affects environmental attitude. As a successful example, 90% of people in Germany actively participate in the waste classification plan due to their improved understanding and attitude toward waste management plans (Schwarz-Herion et al., 2008). Subsequently, more and more scholars began to pay attention to the interrelationship among knowledge, attitude, and behavior (Polonsky et al., 2012; Babaei et al., 2015; Taufique et al., 2016; Baser et al., 2017; Almasi et al., 2019; Khan, 2021). It is worth noting that most of the research on knowledge in the KAB model is single dimensional or incompletely classified (Frick et al., 2004). This leads to the fact that the model cannot fully explain the impact of different forms of environmental knowledge on people's behavior. Moreover, residents' environmental knowledge is closely related to environmental concerns (Tadesse, 2009). This means that simply studying the KAB model cannot fully explain how environmental knowledge affects residents' pro-environmental behavior. Thus, it is necessary to improve the KAB model by adding a new variable.

### Environmental knowledge

Frick et al. (2004) found that most of the research only explores one or at most two kinds of environmental knowledge. This research did not comprehend the comparative effect of various types of knowledge on environmental protection behavior. This negligence leads to a deficiency of research on how various forms of knowledge contribute to environmental protection behavior (Frick et al., 2004). Polonsky et al. (2012) also confirmed that consumers' assessments of different environmental knowledge are not the same and proposed studying different types of environmental knowledge to shape the overall environmental attitude. Apparently, the above examples emphasize the importance of studying multiple types of environmental knowledge.

Ellen (1994) began systematic research as early as the 1990s to consider environmental knowledge as an influential factor in residents' environmental behavior. He distinguished environmental knowledge into subjective and objective knowledge and pointed out that people with more knowledge (both subjective and objective) would be more likely to engage in meaningful pro-environmental behavior. Park et al. (1994) and Dodd et al. (2005) further defined the concepts of these two kinds of knowledge. That is, objective knowledge involves the factual knowledge that has been tested and approved, which helps to organize knowledge and store it in personal memory, and can usually objectively reflect a person's understanding of an object or problem (Park et al., 1994). Subjective knowledge involves one person's view of his (or her) own knowledge, which reveals the individual's self-evaluation and perception of objects, products, or problems (Flynn and Goldsmith, 1999; Carlson et al., 2009). Subsequently, more scholars have also divided environmental knowledge into subjective knowledge and objective knowledge (Carmi et al., 2015; Onel and Mukherjee, 2016; Casaló et al., 2019).

In addition, a large number of researchers have confirmed that subjective knowledge has a more significant effect on individuals' pro-environmental behavior (Kaiser and Fuhrer, 2003; Aertsens et al., 2011; Levine and Strube, 2012; Carmi et al., 2015; Casaló et al., 2019). Therefore, subjective environmental knowledge is worth paying close attention to. This paper focuses on the influence of subjective environmental knowledge on waste sorting intention.

Most of the existing research regards subjective environmental knowledge as a single variable. Kaiser and Fuhrer (2003) and Frisk and Larson (2011) both argued that environmental knowledge is a complex and multifaceted factor rather than a single and coherent one. Therefore, there should be many dimensions of subjective environmental knowledge. According to Frick et al. (2004), there are at least three forms of environmental knowledge: system knowledge, action-related knowledge, and effectiveness knowledge. Braun and Dierkes (2017) also point out that if a person intends to do something beneficial for the environment, he or she has to first know the elementary composition and functional performance of an ecosystem (system knowledge), and then, knowledge related to environmental problem solutions (action-related knowledge) and the benefits of sustainable behavior (effectiveness knowledge) are considered to be the keys to the individual's choice of pro-environmental behavior. In the KAB model, this classification method is consistent with a series of psychological processes of residents in waste sorting. Thus, in this paper, subjective environmental knowledge is distinguished in the KAB model as system knowledge, action-related knowledge, and effectiveness knowledge. According to the above, system knowledge (i.e., procedural knowledge) corresponds to factual or conceptual understanding, in this case, knowledge about ecosystems or information about human impact on the earth (Baierl et al., 2022). For example, in waste sorting, system knowledge usually involves knowledge of all kinds of problems that domestic waste brings to the environment (Kaiser and Fuhrer, 2003). For instance, the ecosystem is facing water, soil, and air pollution, and the mixed disposal of human domestic waste is aggravating the pollution and causing current environmental pressure. If a person thinks that he or she understands the current series of environmental pollution and the causes of such pollution, he or she may take a positive attitude toward environmentally friendly behaviors (such as waste sorting) and will consider that waste sorting is beneficial to reducing environmental pollution. Action-related knowledge refers to the information directly related to waste sorting behavior (Kaiser and Fuhrer, 2003). For example, acquiring action-related knowledge can lead to a better understanding of possible means of settlement to alleviate global water shortages and enable persons to take appropriate action in certain situations (Braun and Dierkes, 2017). Therefore, when people have knowledge of waste sorting methods, they will be likely to consider that they have enough ability to correctly classify waste. This can decrease the perceived difficulties and thus enable them to hold a positive attitude toward waste sorting (Liu et al., 2019). Moreover, action-related knowledge has been shown to be more effective in promoting behavior change than system knowledge (Smith-Sebasto and Fortner, 1994; Kaiser and Fuhrer, 2003). Effectiveness knowledge involves the relative benefits or benefits of waste sorting to the environment (Kaiser and Fuhrer, 2003). Studies have shown that knowledge of effectiveness is essential to achieving a certain behavioral goal (Kaiser and Fuhrer, 2003; Frick et al., 2004). For instance, if a person does not believe that water conservation is environmentally sustainable, this negative attitude may impact his or her behavior (Braun and Dierkes, 2017). In the context of waste sorting, when individuals think they know what benefits waste sorting can bring to the environment, they will more likely hold a positive attitude toward waste sorting. Accordingly, we make the following hypotheses:

H1: System knowledge positively affects the attitude toward waste sorting.
H2: Action-related knowledge positively affects the attitude toward waste sorting.
H3: Effectiveness knowledge positively affects the attitude toward waste sorting.

### Attitude toward waste sorting and waste sorting intention

A key hypothesis of the TRA is that behavioral intention is affected by attitude, leading to the occurrence of behavior (Kumar, 2019). Some studies suggested that environmental behavior intention is directly impacted by attitude toward a particular behavior (Tadesse, 2009; Bortoleto et al., 2012; Polonsky et al., 2012; Singh et al., 2018; Ali et al., 2022). Karim Ghani et al. (2013) also argued that attitude was considered to be the strongest predictive factor of recycling and waste sorting intention. Wang Y. et al. (2020) demonstrated that attitude toward waste sorting positively influences waste sorting intention. Moussaoui et al. (2020) showed that people with higher pro-environmental attitudes behaved in a more environmentally friendly way than those with lower pro-environmental attitudes. In this paper, if people have positive attitudes toward waste sorting, they will form the intention to sort waste. Accordingly, we make the following hypothesis:

H4: Attitude toward waste sorting positively affects waste sorting intention.

### The moderating role of environmental concern

Environmental concern is considered a vital predictor of people's recycling activities (Domina and Koch, 2002; Bamberg, 2003; White and Simpson, 2013; Pagiaslis and Krontalis, 2014; McDonald et al., 2015; Felix et al., 2018). It was initially defined as “whether persons are conscious of environmental issues and whether they are willing to solve these problems, reflecting their attitudes toward environmental protection” (Van Liere and Dunlap, 1981). Later, researchers defined it as “an integrated and general perspective on environmental issues” (Yue et al., 2020). It can be found that these “environmental issues” are related to system knowledge. It also means that environmental concern may have some connection with environmental knowledge to a certain extent. According to Tadesse (2009), post-materialism points out that individual environmental concern is relative to environmental knowledge, but what kind of connection exists between the two needs to be further explored.

Some investigators discovered that environmental concern significantly affects residents' environmental behavior (Ishaswini and Datta, 2011; Pinto et al., 2011). Domina and Koch (2002) showed that it was often the person who cared about the environment and who recycled waste. Ishaswini and Datta's (2011) research shows that environmental concerns positively affect the green purchasing behavior of Indian residents. It can be seen that most of the research on environmental concern focuses on its direct or indirect impact on pro-environmental behavior. Actually, in the field of consumer research, environmental concern has been used as a moderator to study the green purchasing behavior of residents. For instance, Das and Ramalingam (2019) take environmental concern as a moderating variable to study its moderating effect on perceived environmental knowledge and price fairness. This means that the more people pay attention to environmental problems, their perceived environmental knowledge makes them think that the purchase of a green product can bring them greater value, and the more they can perceive the price fairness. From this, it can be speculated whether environmental concern can also be used as a moderating variable in the KAB model.

In addition, some researchers found that environmental knowledge had a weak impact on attitude toward waste sorting in a certain dimension (Braun and Dierkes, 2017; Casaló et al., 2019). At the same time, it has been proven that environmental concerns can positively impact environmental attitudes. For instance, Clark et al. (2003) and Chen and Tung (2014) consider that the more attention consumers pay to the environment, the more positive their attitude toward pro-environmental behavior will be. This means that a person who is highly concerned about the environment believes that environmental issues are more important than other things in life. Therefore, when they perceive their environmental knowledge to be higher, they may possibly take a more positive attitude toward waste sorting. Accordingly, we make the following hypotheses:

H5: Environmental concern positively moderates the relationship between system knowledge and attitude toward waste sorting.
H6: Environmental concern positively moderates the relationship between action-related knowledge and attitude toward waste sorting.
H7: Environmental concern positively moderates the relationship between effectiveness knowledge and attitude toward waste sorting.

**Figure 1:**
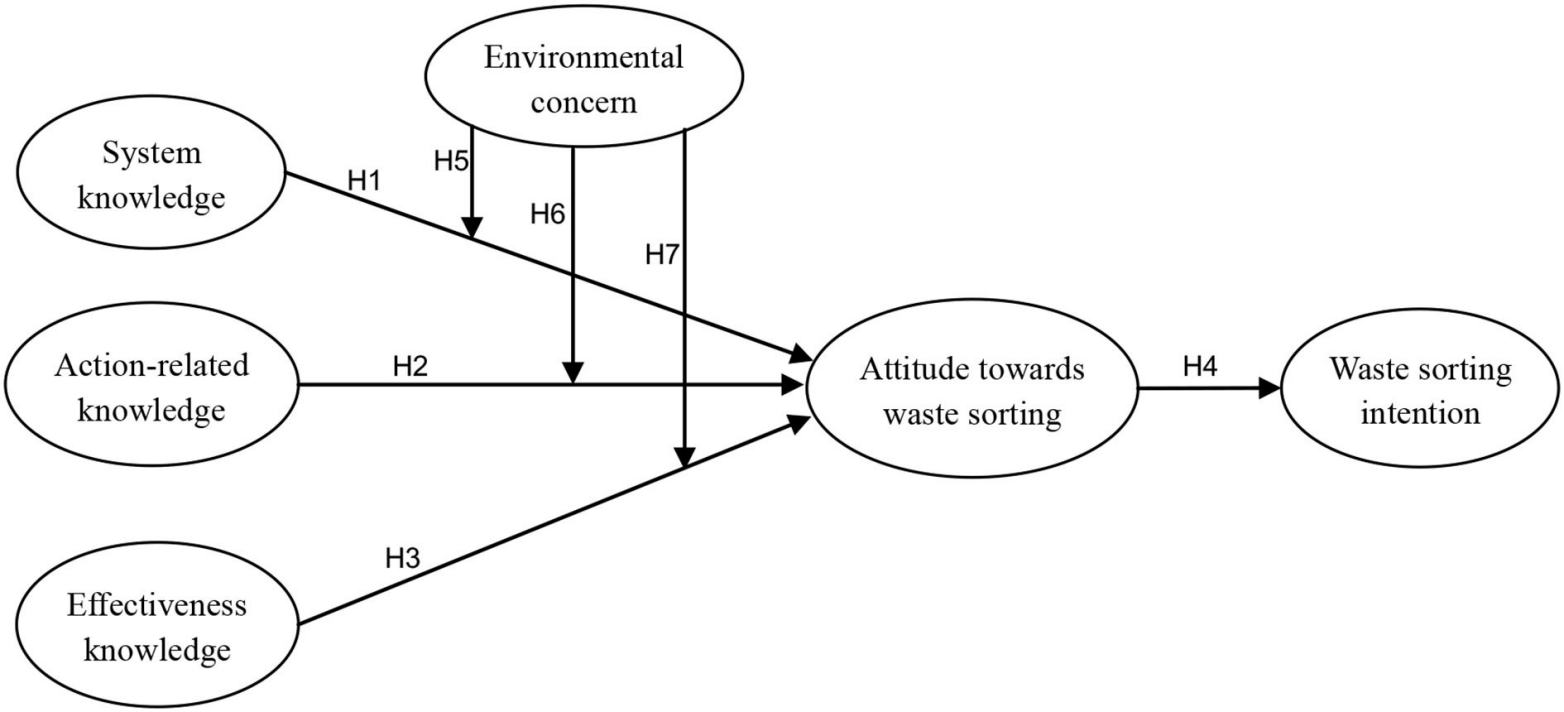
Research model.

Based on the above hypotheses, this article's conceptual model is represented in Figure 1. It describes that the three dimensions of subjective environmental knowledge, namely, system knowledge, action-related knowledge, and effectiveness knowledge, are the antecedents of the attitude toward waste sorting. Environmental concern regulates the relationship between the three kinds of environmental knowledge and the attitude toward waste sorting.

## Methodology

### Questionnaire design

This conceptual model is composed of five variables (see Figure 1). Each variable was measured using a multi-item measurement method (see Table 1), and each item was measured on a five-point Likert scale. For the sake of strengthening the reliability and validity of the study, the items in the questionnaire of this study were obtained from the existing literature (Straub et al., 2004; Kumar, 2019; Wang S. et al., 2020). The questionnaire was written in English at the beginning. As this investigation was conducted in China, two local Chinese doctoral students who are fluent in English first translated all the items into Chinese according to the methods of the translation committee (Vijver and Leung, 1997). At first, our contemporaries and friends filled in the Chinese questionnaire. We finally collected 48 valid questionnaires. Subsequently, for the sake of enhancing the clarity and understanding of the questionnaire, some items were revised based on the comments received.

**Table 1:** Questionnaire items.

**Variable**	**Item**	**Content**	
System knowledge	SK1	Waste sorting can prevent environmental pollution.	Braun and Dierkes (2017)
	SK2	Waste sorting can conserve natural resources.	
	SK3	Waste sorting can improve the quality of the environment.	
Action-related knowledge	AK1	I know what kinds of domestic waste are divided into.	Sorkun (2018)
	AK2	I know how to classify domestic waste.	
	AK3	I know what kind of garbage different kinds belong to.	
Effectiveness knowledge	EK1	Waste sorting helps reduce air pollution.	Wang S. et al. (2020)
	EK2	Waste sorting helps reduce water pollution.	
	EK3	Waste sorting helps reduce soil pollution.	
Environmental concern	EC1	I think environmental problems have become more serious in recent years.	Wang et al. (2017)
	EC2	I think environmental pollutions a threat to me and my family.	
	EC3	I think humans and nature should live in harmony.	
Attitude	AT1	I think waste sorting is useful.	Kumar (2019)
	AT2	I think waste sorting is necessary.	
	AT3	I think waste sorting should be further promoted.	
Waste sorting Intention	WSI1	I am willing to participate in waste sorting.	Wang S. et al. (2020)
	WSI2	I plan to participate in waste sorting.	
	WSI3	I intend to participate in waste sorting.	

### Data collection

The objective groups of this research were a population capable of waste sorting, the survey was conducted between December 20, 2019, and January 10, 2020, and through the way of questionnaire. We chose Chengdu as the research city because Chengdu has a favorable foundation in waste sorting and has carried out domestic waste sorting pilot projects in some residential communities since 2009. In 2017, it was listed as one of China's 46 key cities for the first trial of domestic waste classification. On March 1, 2021, the Regulations of Chengdu on Domestic Waste Management were officially implemented, which means that Chengdu residents officially ushered in the era of waste classification. The main targets of the survey are those who can reach the waste sorting. The scope of the survey includes communities, parks, squares, and other places that can reach different people. Subjects will receive a commemorative pen to participate in the survey after completing the questionnaire. We received a total of 389 completed questionnaires. The answers to each questionnaire were carefully examined and answers with too many missing values were eliminated. Finally, 308 valid questionnaires were obtained. Among these respondents, male respondents accounted for 56.5% and female respondents for 43.5%. Most of the respondents' (83.4%) ages ranged from 20 to 60 years. Fifty percent of the respondents had an educational background of a bachelor's degree or above.

## Results

The research model shown in Figure 1 was analyzed using the partial least squares structural equation modeling (PLS-SEM) method in Smart-PLS 3.0. PLS is suitable for studies with small sample sizes because it does not require multivariate normal distribution (Barclay et al., 1995; Chin, 1998; Hair et al., 2006).

### Measurement model analysis

The reliability and validity of the reflective measurement model should be evaluated (Shanmugapriya and Subramanian, 2016). In this paper, confirmatory factor analysis (CFA) was used to measure it. First, the evaluation criteria were internal consistency reliability. The common internal consistency standard is Cronbach's alpha. It provides reliability estimation based on the correlation of indicators (Streiner, 2003). As given in Table 2, Cronbach's values are higher than the minimum recommended value of 0.7. In consequence, the scale has favorable internal reliability (Rahman et al., 2013). Second, the convergent validity of the model is evaluated. This effectiveness refers to the level at which two or more projects of a particular structure are theoretically interrelated (Campell and Fiske, 1959). Convergence validity was estimated by using factor load on the corresponding structure and average variance extraction (AVE) of the structure (Fornell and Larcker, 1981). Table 2 shows that the values of standardized loading range from 0.717 to 0.959, which are greater than the recommended threshold of 0.7, and the values of AVE range from 0.633 to 0.891, and all exceeded the reference value of 0.50. In addition, the values of CR are between 0.837 and 0.961, all exceeding the specified value of 0.7. The above results prove that the scale has good convergence validity (Gefen et al., 2000).

**Table 2:** Standardized item loadings, AVE, CR, and alpha values.

**Factor**	**Item**	**Standardized loading**	**AVE**	**CR**	**Cronbach's alpha**
System knowledge	SK1	0.865	0.778	0.913	0.857
	SK2	0.908			
	SK3	0.873			
Action-related knowledge	AK1	0.935	0.891	0.961	0.939
	AK2	0.959			
	AK3	0.937			
Effectiveness knowledge	EK1	0.869	0.831	0.936	0.898
	EK2	0.940			
	EK3	0.924			
Environmental concern	EC1	0.717	0.633	0.837	0.718
	EC2	0.858			
	EC3	0.806			
Attitude	AT1	0.883	0.794	0.920	0.870
	AT2	0.915			
	AT3	0.875			
Waste Sorting Intention	WSI1	0.892	0.852	0.945	0.912
	WSI2	0.952			
	WSI3	0.923			

SK, system knowledge; AK, action-related knowledge; EK, effectiveness knowledge; EC, environmental concern; AT, attitude; WSI, waste sorting intention.

Third, discriminative validity means that the observed values should be able to distinguish when measuring different constructs (Campell and Fiske, 1959). In Table 3, the AVE square root of each latent variable is greater than the correlation coefficient of each factor with other factors, which supports the discriminant validity. Therefore, the scale had good discriminant validity.

**Table 3:** The square root of AVE and factor correlation coefficients.

	**SK**	**AK**	**EK**	**EC**	**AT**	**WSI**
SK	**0.882**					
AK	0.228	**0.944**				
EK	0.415	0.226	**0.912**			
EC	0.586	0.101	0.338	**0.796**		
AT	0.474	0.288	0.459	0.380	**0.891**	
WSI	0.334	0.318	0.404	0.216	0.534	**0.923**

The diagonal numbers are the square roots of the mean variance of each construct; Pearson's correlations are shown below the diagonal. SK, system knowledge; AK, action-related knowledge; EK, effectiveness knowledge; EC, environmental concern; AT, attitude; WSI, waste sorting intention.

### Structural model analysis

**Figure 2:**
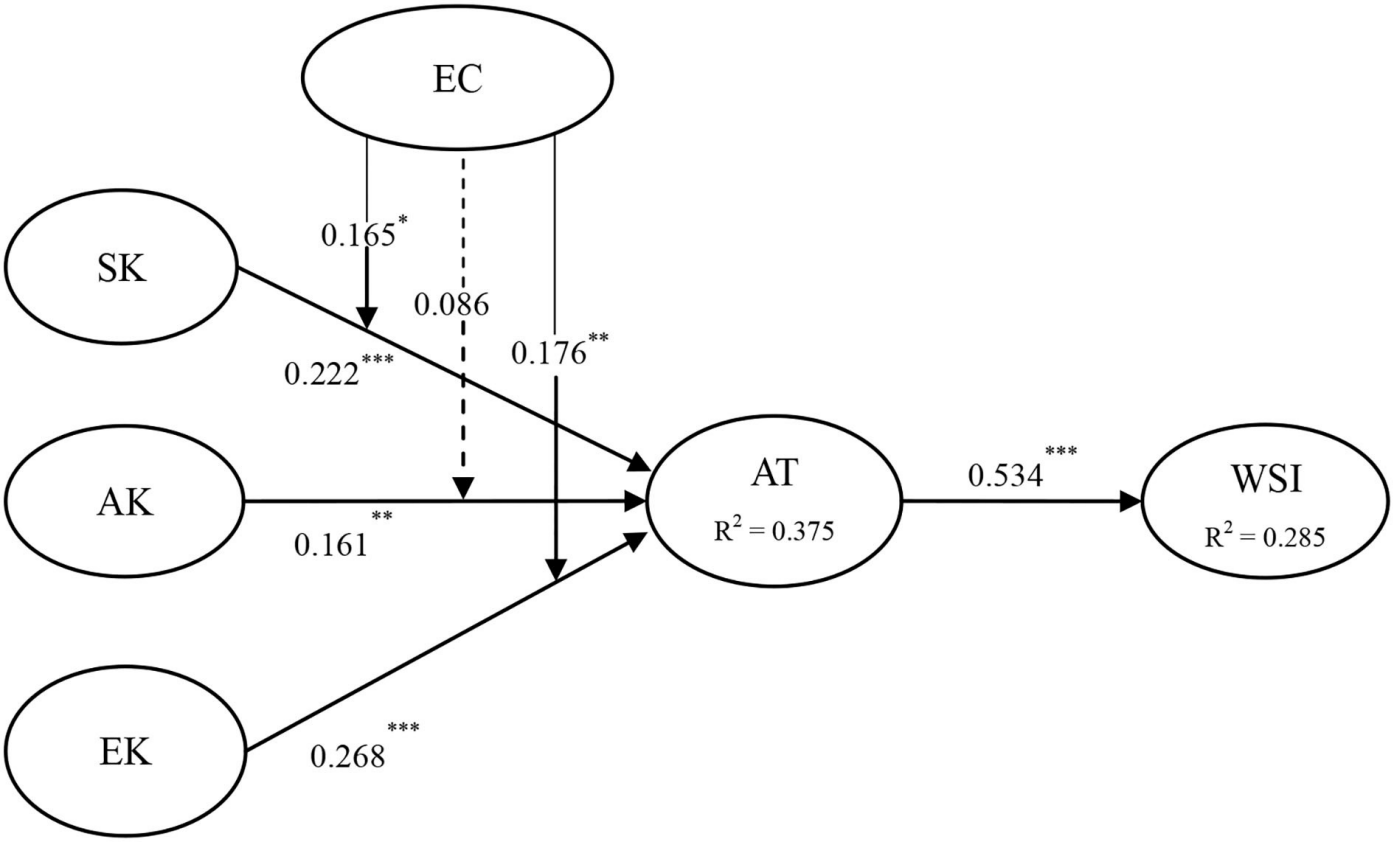
Results of the research model tests. ***p < 0.001; **p < 0.01; *p < 0.05; SK, System knowledge; AK, action-related knowledge; EK, effectiveness knowledge; EC, environmental concern; AT, attitude; WSI, waste sorting intention.

Figure 2 and Table 4 show the analysis results of the structural model. The results provide comprehensive interpretation capabilities, the coefficient of determination (R2) of the endogenous potential variables, the significance of the path coefficient (β value), the relative *t*-value of each path, etc. The bootstrap resampling program is used to verify the significance of the path.

**Table 4:** Hypotheses testing results.

**Hypotheses**	**Structural path**	**Path coefficients**	**T Statistics**	**Supported**
H1	SK->AT	0.222^***^	3.673	Yes
H2	AK->AT	0.161^**^	3.058	Yes
H3	EK->AT	0.268^***^	4.358	Yes
H4	AT->WSI	0.534^***^	11.094	Yes
H5	SK × EC->AT	0.165^*^	2.356	Yes
H6	AK × EC->AT	0.086	0.869	No
H7	EK × EC->AT	0.176^**^	2.500	Yes

*** p < 0.001;

** p < 0.01;

* p < 0.05.

The consequence shows that system knowledge directly and actively affected attitude (β = 0.222, *p* < 0.001). Thus, H1 is supported. Action-related knowledge directly and positively affected attitude (β = 0.161, *p* < 0.01). Consequently, H2 is supported. Furthermore, effectiveness has a direct positive impact on attitude (β = 0.268, *p* < 0.001). Consequently, H3 is supported. Attitude directly and positively affects the waste sorting intention (β = 0.534, *p* < 0.001). Thus, H4 is supported. The model explained 37.5% of the variance related to attitude, and 28.5% of the variance exists in waste sorting intention. Besides, control variables such as age, gender, and education level have no confounding influence on the waste sorting intention. We conducted the multiple regression analysis (Cohen and Cohen, 1983) and simple slope analysis (e.g., to compare the differences between groups with high environmental concerns and groups with low environmental concerns) to explore the moderating effects of environmental concerns on the relationship between system knowledge, action-related knowledge, effectiveness knowledge, and attitude toward waste sorting. First, the EC value is multiplied by the values of three antecedent variables to establish three regulatory products. Second, the antecedent variables, moderating factor, and product terms were added to the regression model. Significant product terms will prove that EC has a moderating effect. As given in Table 4, EC can significantly and positively moderate the effect of effectiveness knowledge on attitude at the *P* < 0.01 level. Thus, H7 is supported. In addition, EC played a significant positive moderating role in the impact of system knowledge on attitude at *P* < 0.05 level. Therefore, H5 is supported. However, EC does not moderate the effect of action-related knowledge on attitude. Thus, H6 is not supported.

**Figure 3:**
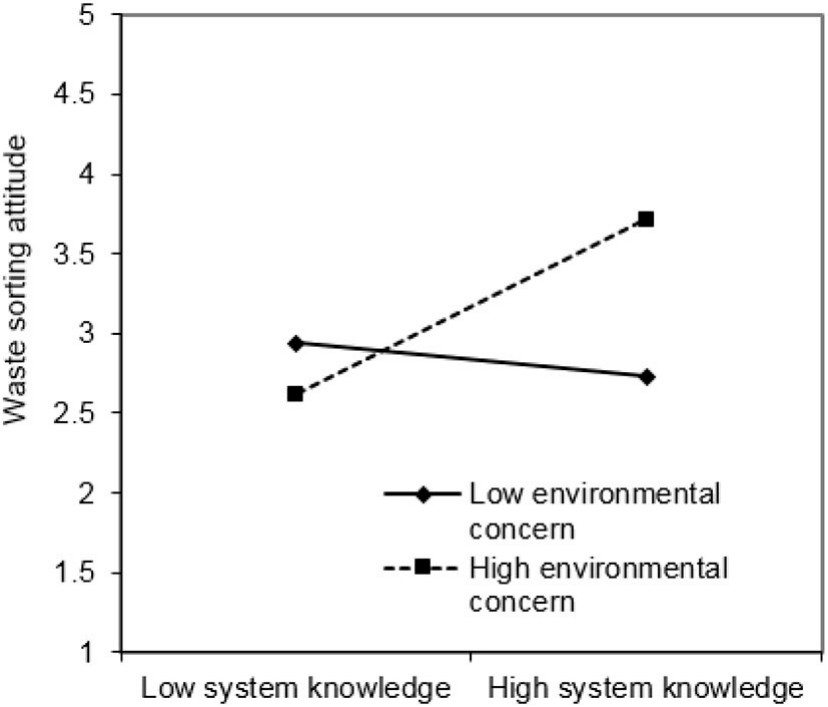
Moderation plot of system knowledge.

**Figure 4:**
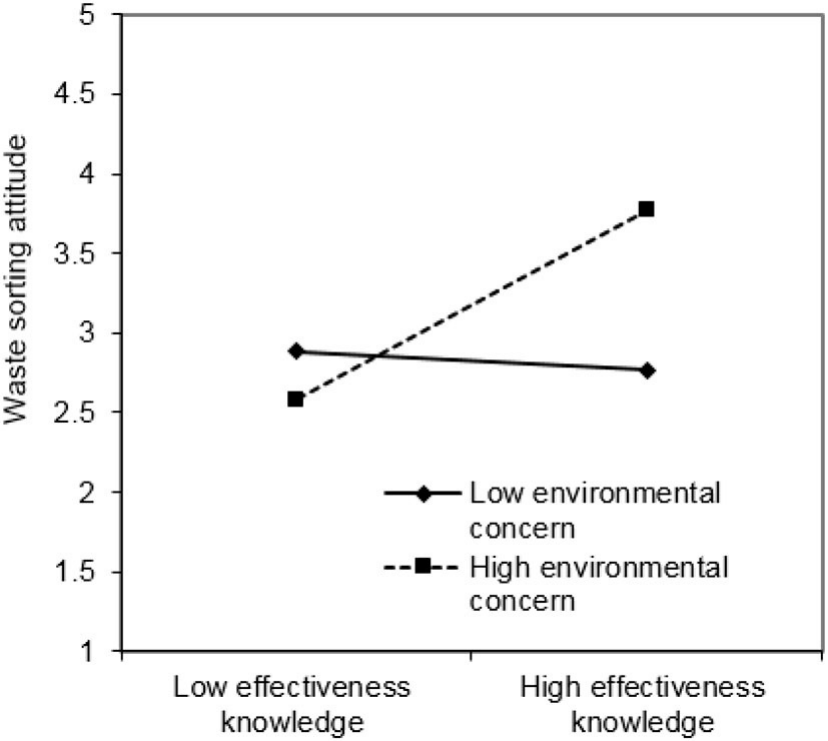
Moderation plot of effectiveness knowledge.

Additionally, this paper employed the simple slope test (Cohen and Cohen, 1983; Aiken and West, 1991). The regression lines that are one standard deviation higher than the average value of environmental concern and one standard deviation lower than the average value of environmental concern were tested. Figures 3, 4 show the relationship between attitudes toward waste sorting and environmental knowledge under high and low environmental concern, respectively.

## Discussion

The study is designed to explore the influencing factors of urban residents' waste sorting behavior intention. To achieve this goal, we added three different forms of subjective environmental knowledge as antecedents of attitude toward waste sorting. In the meantime, based on the KAB model, environmental concern was added to moderate the effects of the three types of knowledge on waste sorting attitudes. In this study, an extended KAB model was proposed and tested to successfully predict the behavioral intention of household waste sorting.

First, the current research states that the three different forms of subjective environmental knowledge are positive prerequisites for the formation of residents' attitudes toward waste sorting. That is to say, three types of subjective environmental knowledge all have significant positive effects on residents' attitudes toward waste sorting, namely, H1, H2, and H3. That shows no difference from the previous findings of Flamm (2009) and Polonsky et al. (2012). This indicates that when residents have a higher awareness of different types of environmental knowledge, they have a more positive attitude toward waste sorting, thus showing a greater waste sorting intention. Our results also found that residents' self-reported effectiveness knowledge has the most significant influence on attitude toward waste sorting, while action-related knowledge has a less significant correlation with attitude toward waste sorting than the other two types of knowledge. This finding is not consistent with that of Kaiser and Fuhrer (2003), which shows that action-related knowledge can promote behavior change more effectively than systemic knowledge. Similarly, Frick et al. (2004) indicate that effectiveness knowledge is vital for the realization of a behavioral goal. With this form of knowledge, the focus on action-related knowledge has obviously been extended from a mere knowing how to conserve to knowing how to get the greatest environmental benefit (Hanna, 2010). This finding also reflects the current state of society in which people may be more concerned with the consequences of a particular action than with the cause or process. In addition, these results are consistent with the current policies of the government. For instance, in order to further popularize the knowledge of household waste sorting to the residents and publicize the benefits of waste classification, the Ministry of Housing and Urban–Rural Development and the China Government Network jointly launched the “national garbage classification” small program. This will help to increase people's environmental knowledge related to waste classification, so as to improve the public's attitude toward waste classification.

Second, the consequences of current research indicate that residents' attitude toward waste sorting positively and significantly affects their household waste sorting behavior intention (H4). This result also confirms the key hypothesis in the TRA that attitude has a correlation with behavioral intention (Kumar, 2019) and is in line with the findings of Singh et al. (2018), Bortoleto et al. (2012), and Tadesse (2009). In other words, environmental behavior is likely to be directly affected by attitude toward specific behavior. This reflects the intuition that it is difficult for householders to classify waste if they do not have a positive view of the behavior and its potential consequences (Ma et al., 2018). Thus, it is suggested that in the goal of promoting households' intention/behavior in waste sorting, emphasis should be put on strategies that reinforce residents who have a more positive attitude and, on the other hand, change those with a negative one.

Finally, the results of this study show that environmental concern moderates the relationships between system knowledge, effectiveness knowledge, and attitude toward waste sorting. Environmental concern plays a positive moderating role in the impact of system knowledge on attitude toward waste sorting (H5). This is identical with the previous research results of Clark et al. (2003) and Chen and Tung (2014). It signifies that if people are more worried about environmental problems, the impact of system knowledge on attitudes toward waste sorting will be more significant. Environmental concern will make the residents more likely to recognize the severity of environmental problems and the importance of waste sorting, thus changing their attitudes toward waste sorting. Similarly, environmental concern plays a positive moderating role in the impact of effectiveness knowledge on attitude toward waste sorting (H7). This means that residents who care about the environment are more conscious of the environmental benefits of waste sorting, so they will have a positive attitude toward waste sorting. However, H6 is not supported, that is, environmental concern has no moderating effect on action-related knowledge and attitude toward waste sorting. The probable cause for this result is that action-related knowledge refers to information directly related to actions, but not the consequences, while environmental concern refers to people's concern about environmental consequences (Lee, 2008). Even though one does not pay much attention to environmental issues, he will take a positive attitude toward waste sorting if he knows how to do it. On the contrary, even if an individual is very concerned about environmental problems, but does not know how to carry out waste sorting, he or she may also have a negative attitude toward waste sorting because of a lack of specific operational knowledge. This result also reflects the current situation of waste sorting in China. According to the 2019 Survey on the Environmental Awareness of Chinese Urban Residents released by Shanghai Jiao Tong University, the number of respondents who keep a positive attitude toward waste sorting exceeded 90%, because they think it helps environmental protection. However, most of them are not familiar with the knowledge of waste sorting, and in the actual operation process of waste sorting, their spirit is willing but the flesh is weak, thus giving up waste sorting (You, 2020).

## Research contribution

### Theoretical implications

In summary, this paper makes several important theoretical contributions for enriching the existing research on residents' waste sorting behavior. First, a comprehensive theoretical model is constructed based on the KAB model to explore the influence mechanism of different dimensions of subjective environmental knowledge on urban residents' garbage classification intention. On the one hand, it enriches the theoretical framework of current research on waste sorting. On the other hand, it also provides a research framework for other studies in the field of pro-environmental behavior.

Second, this paper studies the role of different forms of subjective knowledge. Although many researchers have discussed the influence of subjective environmental knowledge on people's pro-environmental behaviors, they studied it from a single dimension (Kaiser and Fuhrer, 2003; Aertsens et al., 2011; Levine and Strube, 2012) and have not considered studying its role from different dimensions. This study attempts to divide subjective environmental knowledge into system knowledge, action-related knowledge, and effectiveness knowledge, and successfully verifies the positive effect of the three types of subjective environmental knowledge on the behavioral intention of waste sorting through residents' attitudes toward waste sorting. Tang et al. (2022) also proved that environmental knowledge plays a positive role in encouraging and promoting residents' domestic waste classification behavior. In addition, this study also proves that compared with the other two kinds of subjective environmental knowledge, effectiveness knowledge has the most significant impact on residents' attitude toward waste sorting, while action-related knowledge has the lowest significance. Finally, the moderating effect of environmental concerns is confirmed in this study. Most of the previous studies only discussed the direct or indirect influence of environmental concerns on residents' pro-environmental behaviors (Domina and Koch, 2002; Ishaswini and Datta, 2011; Pinto et al., 2011), and few studies took environmental concerns as a moderator variable to study pro-environmental behaviors. In addition to expanding the research of Tadesse (2009), this study also proves the moderating role of environmental concern. In other words, individuals who attach great concern to the environment are more likely to be motivated by system and effectiveness knowledge and show a positive attitude toward waste sorting, thus generating greater behavioral intention of waste sorting.

### Managerial implications

There are several practical implications of this study. First, the relevant departments should pay attention to the effect of environmental knowledge when publicizing waste sorting. This study shows that when the residents think that they do not know the environment, they are likely to have a negative attitude toward waste sorting. At present, it is understood that relevant government departments have issued waste sorting manuals to the community to publicize how to carry out waste sorting. Relevant departments in Chengdu have also released WeChat programs for waste sorting. It follows that the publicity related to waste sorting only attaches importance to the popularization of action-related knowledge. Thus, while strengthening the publicity of action-related knowledge, the government should pay equal attention to the publicity of system knowledge and effectiveness knowledge. For example, this type of knowledge can be publicized through traditional media (one-way communication media such as newspapers, radio, and TV), interactive media (digital media such as WeChat and Weibo), life-circle media (such as shopping malls, amusement parks, and other offline terminals), and other channels. The specific content of the promotion of system knowledge and effectiveness knowledge can be focused on the environmental hazards of not separating garbage (system knowledge) and the environmental benefits of garbage separation (effectiveness knowledge). Meanwhile, according to the results of this study, effectiveness knowledge has the most significant impact on residents' attitudes toward waste sorting. Therefore, when publicizing environmental knowledge in the main media, the government should focus on effective knowledge. Intuitive materials can be used to publicize the benefits of waste sorting. It is necessary to emphasize the importance of waste sorting in promoting the circular economy and coping with global warming. It is also necessary to publicize and educate residents about the risks related to climate change, so that residents can truly feel that waste sorting is not only an altruistic act but also a self-interest act in the long run.

Second, the government is supposed to focus on cultivating residents' environmental concern when propagating environmental knowledge. This study concludes that the subjective environmental knowledge (system knowledge and effectiveness knowledge) possessed by people who have a high degree of environmental concern is more conducive to their positive attitudes toward waste sorting. Consequently, the government can strengthen people's belief in waste sorting by publicizing the concept of environmental protection. Meanwhile, the government should incorporate environmental education into the education system and set up environmental education-related courses in the curriculum of students at various stages so as to cultivate people's environmental concern from childhood.

Third, the government should strengthen the sense of responsibility and the cultivation of values in the residents' waste sorting. The results of this study show that attitude toward waste sorting is a significant forecast factor of waste sorting intention. Consequently, the government department should emphasize improving the residents' attitudes toward waste sorting. This can be done by cultivating public's sense of value and responsibility for waste sorting, especially its environmental and ecological value, energy protection value, and climate value. Thereby, residents gradually develop a relatively stable emotional tendency toward waste sorting.

## Limitations and future research directions

Nevertheless, there are also some limitations in this study. First, the scope of the survey is limited to Chengdu. The investigation of the environmental knowledge status of people may be one-sided due to the difference in education levels between people in different provinces. The situation in other regions is unclear. These issues should be explored in future work. Therefore, further investigation is needed in different provinces in China to improve the generalizability and applicability of the research results. Second, although the survey was conducted by questionnaire, the results are only transverse and the dynamic processes of different periods are not examined. Future follow-up research can be more longitudinal and improve the relationship between variables. Third, this paper does not study the intermediary role of waste sorting attitude between different dimensions of environmental knowledge and waste sorting intention, which can be studied in future. Finally, some researchers believe that the premise of environmental knowledge stimulating environmental behavior is the stimulation of environmental emotion (Carmi et al., 2015). Therefore, the mediating role of environmental emotion can be considered in future research on household waste sorting behavior. Meanwhile, due to the close relationship between knowledge and education, future research can consider the moderating effect of education level. In spite of the limitations mentioned above, this study still further understands the prerequisites for residents' waste sorting behavior and provides effective guidelines for policymakers.

## Data availability statement

The original contributions presented in the study are included in the article/supplementary material, further inquiries can be directed to the corresponding authors.

## Ethics statement

Ethical review and approval was not required for the study on human participants in accordance with the local legislation and institutional requirements. Written informed consent from the patients/participants or patients/participants legal guardian/next of kin was not required to participate in this study in accordance with the national legislation and the institutional requirements.

## Author contributions

YL: research questions, research design, and model building. ZH and HZ: research design, model building, and data collection. FW: data collection and manuscript writing. XL: revision of the paper. All authors contributed to the article and approved the submitted version.

## Funding

This work was supported by Sichuan Center for Rural Development Research (CR2112); Sichuan Science and Technology Plan (2021JDR0340).

## Conflict of interest

The authors declare that the research was conducted in the absence of any commercial or financial relationships that could be construed as a potential conflict of interest.

## Publisher's note

All claims expressed in this article are solely those of the authors and do not necessarily represent those of their affiliated organizations, or those of the publisher, the editors and the reviewers. Any product that may be evaluated in this article, or claim that may be made by its manufacturer, is not guaranteed or endorsed by the publisher.
